# FDG-PET/CT-based respiration-gated lung segmentation and quantification of lung inflammation in COPD patients

**DOI:** 10.1186/s13104-024-06820-w

**Published:** 2024-06-20

**Authors:** Ayse Dudu Altintas Dogan, Thomas Quist Christensen, Torben Tranborg Jensen, Claus Bogh Juhl, Ole Hilberg, Else-Marie Bladbjerg, Søren Hess

**Affiliations:** 1 Department of Medicine, Regional Hospital Horsens, Sundvej 30, 8700 Horsens, Denmark; 2 Department of Medicine, Hospital South West Jutland, University Hospital of Southern Denmark, Esbjerg, Denmark; 3 Department of Regional Health Research, Faculty of Health Sciences, University of Southern Denmark, Esbjerg, Denmark; 4 Department of Medicine, Lillebaelt Hospital, University Hospital of Southern Denmark, Vejle, Denmark; 5 Department of Radiology and Nuclear Medicine, Hospital South West Jutland, University Hospital of Southern Denmark, Esbjerg, Denmark; 6 Steno Diabetes Center, Odense, Denmark; 7 Department of Clinical Diagnostics, Unit for Thrombosis Research, Hospital South West Jutland, University Hospital of Southern Denmark, Esbjerg, Denmark; 8 Department of Clinical Engineering, Region of Southern Denmark, Esbjerg, Denmark; 9 IRIS – Imaging Research Initiative Southwest, Esbjerg, Denmark; 10 Department of Nuclear Medicine, Odense University Hospital, Odense, Denmark

**Keywords:** FDG-PET/CT, COPD, GLP-1 RA, Respiration-gated, Inflammation

## Abstract

**Objective and results description:**

The study objective was to investigate the potential of quantitative measures of pulmonary inflammation by [18 F]Fluorodeoxyglucose positron emission tomography/computed tomography (FDG-PET/CT) as a surrogate marker of inflammation in COPD. Patients treated with anti-inflammatory Liraglutide were compared to placebo and correlated with inflammatory markers. 27 COPD-patients (14 receiving Liraglutide treatment and 13 receiving placebo) underwent 4D-respiratory-gated FDG-PET/CT before and after treatment. Two raters independently segmented the lungs from CT images and measured activity in whole lung, mean standard uptake values (SUVmean) corrected for lean-body-mass in the phase-matched PET images of the whole segmented lung volume, and total lesion glycolysis (TLG; SUVmean multiplied by volume). Inter-rater reliability was analyzed with Bland-Altman analysis and correlation plots. We found no differences in metabolic activity in the lungs between the two groups as a surrogate of pulmonary inflammation, and no changes in inflammation markers. The purpose of the research and brief summary of main findings. The degree of and changes in pulmonary inflammation in chronic obstructive pulmonary disease (COPD) may be difficult to ascertain. Measuring metabolic activity as a surrogate marker of inflammation by FDG-PET/CT may be useful, but data on its use in COPD including reproducibility is still limited, especially with respiration-gated technique, which should improve quantification in the lungs. We assessed several quantitative measures of metabolic activity and correlated them with inflammation markers, and we assessed reproducibility of the methods. We found no differences in metabolic activity between the two groups (before and after 40 weeks treatment with Liraglutide vs. placebo). Bland-Altman analysis showed good agreement between the two raters.

**Trial registration:**

The study was conducted between February 2018 and March 2020 at the Department of Pulmonary Diseases at Hospital South West Jutland and Lillebaelt Hospital, Denmark, and registered from March 2018 at clinicaltrials.gov with trial registration number NCT03466021.

## Introduction

[^18^F]-fluorodeoxyglucose positron emission tomography computerized tomography (FDG-PET/CT) is a well-established molecular imaging technique with an increasing role in infectious and inflammatory diseases. It assesses glucose metabolism as a surrogate marker of disease activity on the molecular level [1].

Chronic obstructive pulmonary diseases (COPD) affect millions of people worldwide with chronic inflammation in airway and lungs, airway limitation and significant morbidity and healthcare utilization. Inflammation plays a significant role, and quantification of inflammatory markers are essential in stable phases and during exacerbations in COPD [2].

Glucagon-Like-Peptide 1 receptor agonists (GLP-1 RA) are used in treatment of diabetes type 2 and for the purpose of weight loss. Among other tissues, GLP-1 receptors are expressed in the lungs and exhibit anti-inflammatory properties by reducing circulating inflammatory markers thereby reducing COPD morbidity and mortality in mice and among patients, GLP-1 RA reduce respiratory diseases including COPD exacerbations [3, 4].

Objective and non-invasive assessment of response to medical treatment in inflammatory diseases may be challenging. FDG-PET/CT is already widely employed to assess inflammation in clinical settings and response evaluation in oncology, but there are only few results regarding response evaluation in inflammatory diseases. However, earlier studies did assess the use of FDG-PET/CT to access inflammation at various stages or to differentiate various subtypes of COPD, but studies were small and exploratory. To the best of our knowledge, this is the first RCT with a well-defined patient population with FDG-PET/CT to assess the effect of intervention.

The rationale to employ respiratory gating is the inherent challenges with movement in the lung region during the long acquisition times of PET/CT. Especially the high activity in the liver could influence the overall quantification of the expected low and diffuse lung uptake if the motion during respiration is not accounted for. Further by applying 4D-respiratory-gated PET/CT we assured alignment of CT and PET during the complete respiratory cycle, which results in improved attenuation and scatter correction and that the delineation of the lungs from CT images would accurately be transferrable to quantify lung uptake in the PET images. All of this resulted in a more robust quantification of lung FDG uptake.

We aimed to investigate if respiration gated quantitative FDG-PET/CT measures, as surrogate for pulmonary inflammation, as well as markers of systemic inflammation are reduced in patients with COPD treated with GLP-1 RA for 40 weeks. Further, we assessed the reproducibility of the FDG-PET/CT measures.

## Materials & methods

We conducted a prospective, randomized, placebo-controlled, double blinded, two-center, parallel-group trial between February 2018 and March 2020 at The Department of Medicine, Section of Pulmonary Diseases, Esbjerg Hospital and Lillebaelt Hospital, University Hospital of Southern Denmark, Denmark.

We randomized 40 obese participants with COPD for treatment with Liraglutide 3.0 mg per day or placebo in a 1:1 manner and followed them for 44 weeks as previously described [5]. We included people with COPD defined as forced expiratory volume in one second relative to forced vital capacity (FEV1/FVC) < 70% after maximal bronchodilation in accordance with Global Initiative for Chronic Obstructive Lung Disease guidelines.

Participants were former smokers with 20 or more pack-years history of smoking and were 40–75 years of age. BMI above 27 kg/m2 was defined as inclusion criteria.

Exclusion criteria were treatment with systemic corticosteroids; diabetes mellitus of any type; interstitial pulmonary disease; asthma or asthma-COPD Overlap Syndrome (ACOS), severe hepatic, renal, or heart disease; history of pancreatitis; pregnancy or breastfeeding. As part of the study setup, we performed an FDG-PET/CT of the thorax at baseline (scan 1) and at end of medication at week 40 (scan 2) to assess any changes in pulmonary tracer uptake as a marker of inflammatory activity. Blood samples were assessed for inflammatory markers at baseline and after 40 weeks. We also conducted scans in three healthy controls and two patients with clinical COPD exacerbation.

FDG-PET/CT was performed according to department protocol based on EANM guidelines, i.e. patients fasted for at least 6 h prior to administration of a weight-adjusted dose of 4 MBq/kg FDG (min. 200 MBq-max. 400 MBq). Plasma glucose levels were routinely measured with an allowed maximum of 8 mmol/L (150 mg/dL). Time between injection and PET/CT acquisition was within 60 +/- 5 min. The 4D-respiratory-gated FDG-PET/CT was performed on a Discovery 710 (GE Healthcare, Milwaukee, Wisconsin, USA) using the real-time position management (RPM) respiratory gating system (Varian Medical Systems Inc., Palo Alto, CA) to monitor the participant’s respiration during acquisition.

Following the CT scan, a PET acquisition was performed over the same lung area comprised of two or three bed positions with 6 min. pr. bed and a slice overlap of 16 slices (34%) with scan field of view of 70 cm saved into list-mode files. Corrections for attenuation, randoms, deadtime, normalization and scatter were performed inside the iterative loop.

After PET reconstructions, the individual phases were summed into a single respiratory phase, using the Q.Freeze 1.0 algorithm. The best alignment between CT and PET images was ensured.

Analysis with regard to quantitative measurements were carried out using a GE Advantage Server 2.0 (GE Healthcare, Milwaukee, Wisconsin, USA). The analysis comprised segmenting both lungs by first applying a threshold with a maximum value of -600 Hounsfield units and manually masking out sections in the threshold that was not part of the lungs. The whole segmented lungs were then transferred as a VOI to the PET series, and the mean activity concentration was extracted. From this activity concentration, standard uptake values (SUVs) were calculated as SUV corrected for body weight (SUVbw) and SUV corrected for lean body mass (SUL). We calculated Total Lesion Glycolysis (TLG) normalizing mean SUVs for lung volume. A nuclear medicine specialist (SH) assessed all PET/CT scans visually.

In this part of the study, our aim was to quantify disease activity in the lungs at baseline and after treatment with Liraglutide 3.0 mg in terms of SUL, SUVbw and TLG. We measured systemic inflammation using the markers C-reactive protein (CRP) (Cardiophase hsCRP, Siemens Healthcare Diagnostic Products, Germany), interleukin-6 (IL-6), and monocyte chemoattractant protein-1 (MCP-1) (IL-6 and MCP-1 Quantikine ELISA kits, R&D Systems, UK).

To validate our findings from the scans, we performed the same gating and segmentation procedures in three controls, i.e. patients with no known pulmonary disorders. Finally, we performed segmentation, but not gating, in two patients with well-known exacerbation of COPD.

To investigate the reproducibility of lung segmentation and measures of pulmonary metabolic activity, a medical doctor (AD) and a physicist (TC) blinded to all information independently performed segmentation of the lungs in all study participants at scans 1 and 2. For analysis of inter-reader reliability Bland-Altman plots with 95% limits of agreement (LOA) and coefficient of variation (CV) where generated along with correlation plots with linear fit and calculated Pearson squared correlation coefficient (r^2^) and sum of squared error (SSE). We used Wilcoxon rank sum test for statistical analysis and random effect models for calculating average group differences. The level of significance was < 5%.

## Results

Of 40 participants, 27 completed the study with both scans; 14 in the Liraglutide arm and 13 in the placebo arm. Baseline group characteristics including anthropometrics, lung function, lung volumina, disease burden and morbidity are listed in Table 1. For further clinical results from the study, please consult [5].

**Table 1:** Baseline characteristics (mean ± SD) in Liraglutide and placebo groups

Group	Liraglutide 3.0 mg (*N* = 14)	Placebo (*N* = 13)
Age (years)	64.8 ± 8.4	65.3 ± 6.7
Gender (male/female)	9/4	9/5
Weight (kg)	104.4 ± 13.6	102.1 ± 18.0
BMI (kg/m^2^)	35.1 ± 3.7	36.6 ± 5.6
FEV1/FVC (%)	57.6 ± 9.4	48.2 ± 11.9
FEV1 (L)	1.8 ± 0.67	1.4 ± 0.67
FEV1 (%)	62.8 ± 17.8	42.8 ± 11.9
FVC (L)	3.16 ± 1.01	2.83 ± 0.99
FVC (%)	86.4 ± 19.1	81.5 ± 19.3
TLC (%)	106 ± 18	114 ± 22
DLCO (%)	67.3 ± 25.1	52.2 ± 24.3
RV (%)	147 ± 48	176 ± 53
MRC dyspnea scale	2.05 ± 1.08	3 ± 1.06
Number of exacerbations the year before inclusion	0.56 ± 0.70	0.77 ± 0.90
CCI	3.26 ± 1.10	3.26 ± 1.05
CAT-score	15.4 ± 6.1	17.8 ± 5.8
6-min walking distance (m)	419 ± 91	308 ± 138

Data are the mean ± SD. Sex is given in numbers. BMI: body mass index; FEV1: forced expiratory volume in one second; FVC: forced vital capacity; TLC: total lung capacity; DLCO: diffusion capacity of the lung for carbon monoxide; RV: residual volume; MRC: Medical Research Council dyspnea scale; CCI: Charlson Comorbidity Index; CAT-score: COPD assessment test score

We calculated results for differences between the Liraglutide and placebo group in measurements of SUL, SUVbw, and TLG. We observed no differences between baseline values of SUL, SUVbw, and TLG. At week 40, SUL was significantly higher in the Liraglutide group than in the placebo group, Table 2.

**Table 2:** Values of the FDG-PET/CT measures SUL, SUVbw, and TLG (mean ± SD) at baseline (scan 1) and after end of treatment week 40 (scan 2)

Variable	Liraglutide (*N* = 14) baseline	Placebo (*N* = 13) baseline	*p*-value baseline	Liraglutide (*N* = 14) Week 40	Placebo (*N* = 13) Week 40	*p*-value week 40
SUL_1_	0.31 ± 0.07	0.25 ± 0.06	NS	0.31 ± 0.07	0.26 ± 0.06	0.04
SUL_2_	0.31 ± 0.07	0.26 ± 0.06	NS	0.31 ± 0.07	0.26 ± 0.06	0.04
SUVbw_1_	0.49 ± 0.10	0.43 ± 0.10	NS	0.49 ± 0.10	0.43 ± 0.09	NS
SUVbw_2_	0.49 ± 0.10	0.43 ± 0.10	NS	0.49 ± 0.10	0.43 ± 0.10	NS
TLG_1_	899 ± 290	952 ± 177	NS	898 ± 279	941 ± 189	NS
TLG_2_	938 ± 291	998 ± 197	NS	935 ± 296	986 ± 205	NS

SUL, SUVbw and TLG obtained independently by rater 1 (suffix 1) and rater 2 (suffix 2). SUL: Standard Uptake Value corrected for lean body mass; SUVbw: Standard Uptake Value corrected for body weight; TLG: Total Lesion Glycolysis

Using mixed effect models, we estimated the effect of treatment on FDG-PET/CT parameters at week 40, separately for both raters. We calculated average group differences, defined as the difference between the average value of a measure in the Liraglutide group and the average value of the same measure in the placebo group at week 40 (scan 2).

We found no significant differences in SUL, SUVbw or TLG between the Liraglutide and placebo group after treatment (all *p*-values above 0.3). Results are given in Table 3.

**Table 3:** Average baseline-adjusted group differences (Liraglutide – placebo) in SUL, SUVbw and TLG

Variable	Results at week 40 by rater 1	*p*-value	Results at week 40 by rater 2	*p*-value
SUL	0.009	0.6	0.007	0.6
SUVbw	0.02	0.3	0.02	0.4
TLG	-12.1	0.8	-17.6	0.8

SUL: Standard Uptake Value corrected for lean body mass; SUVbw: Standard Uptake Value corrected for body weight; TLG: Total Lesion Glycolysis

**Fig. 1:**
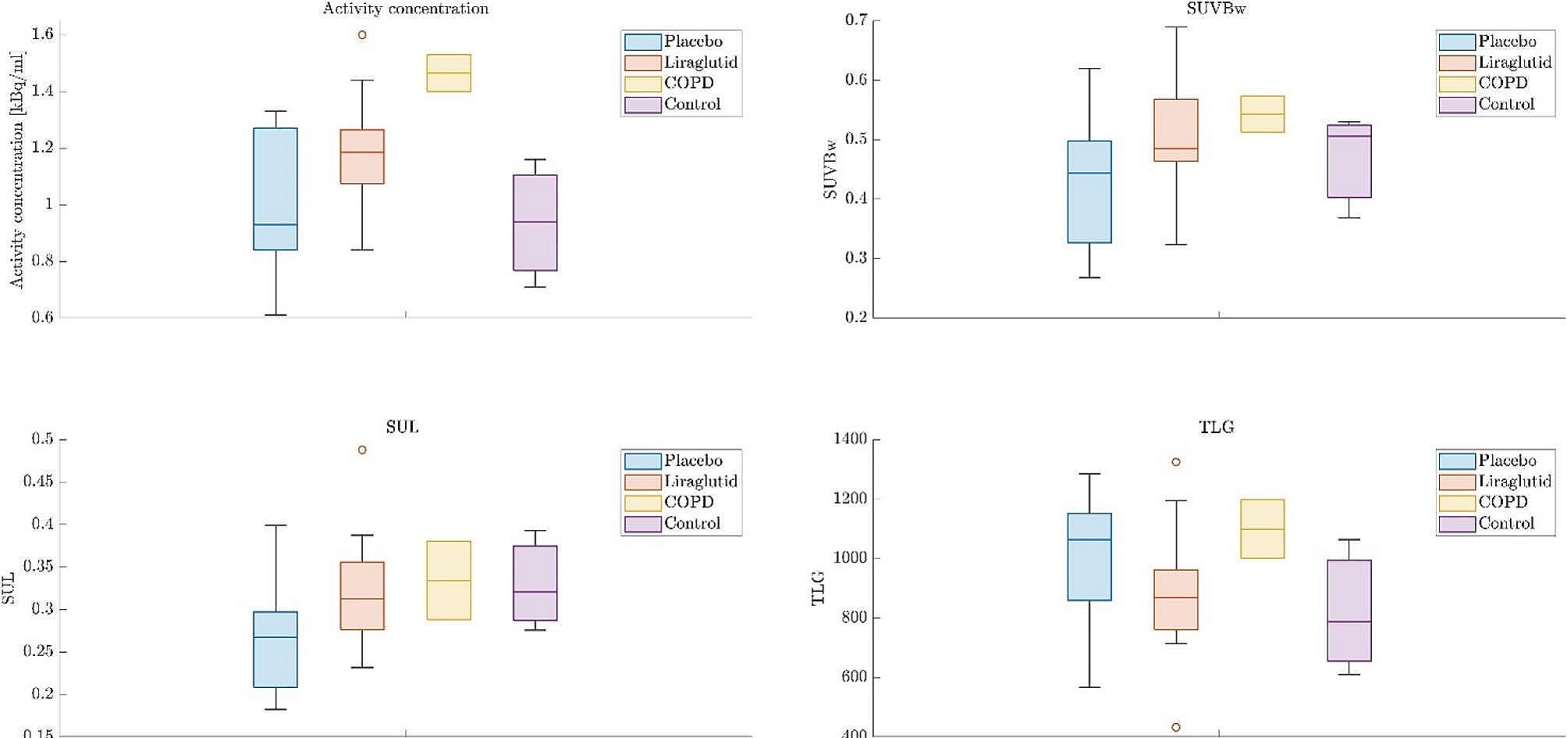
Results for activity concentration, SUVbw, SUL, and TLG for Liraglutide (*N* = 17) and placebo (*N* = 13) (scan 2) for COPD exacerbations (*N* = 2) and controls (*N* = 3) (no known pulmonary disease)

Figure 1 summarizes results for activity concentration, SUVbw, SUL, and TLG for Liraglutide and placebo groups at scan 2 and for COPD exacerbations and controls. We found no significant differences in any PET parameters when comparing the three controls to either Liraglutide or placebo groups. When we compared the results from the two patients with clinical COPD exacerbation to controls and the Liraglutide or placebo groups, we found a tendency towards higher values in patients with clinical COPD exacerbations. Results were most pronounced for overall tracer uptake and less pronounced for median values of SUVbw, SUL, and TLG.

When the uncorrected activity concentration were measured in the different groups, only the COPD group displayed a different and increased uptake value. When adjusting for the decay corrected injected activity and patient weight in SUVBw and SUL, this increased value in the COPD group was not found and measurements between groups was not significantly different although the placebo group had a tendency of lower values. Especially when adjusting for lean body mass in the SUL measurements the Liraglutide, COPD and control groups were almost identical, which was probably due to its correction for a non-equal distribution of male and female participants in the COPD and control group.

The TLG measurements took into consideration the total segmented lung volume of the measurements, which on average was found to be different for the different groups. However, this does not seem to be a reliable indicator of decease progress as no significant differences was found between the groups.

**Fig. 2:**
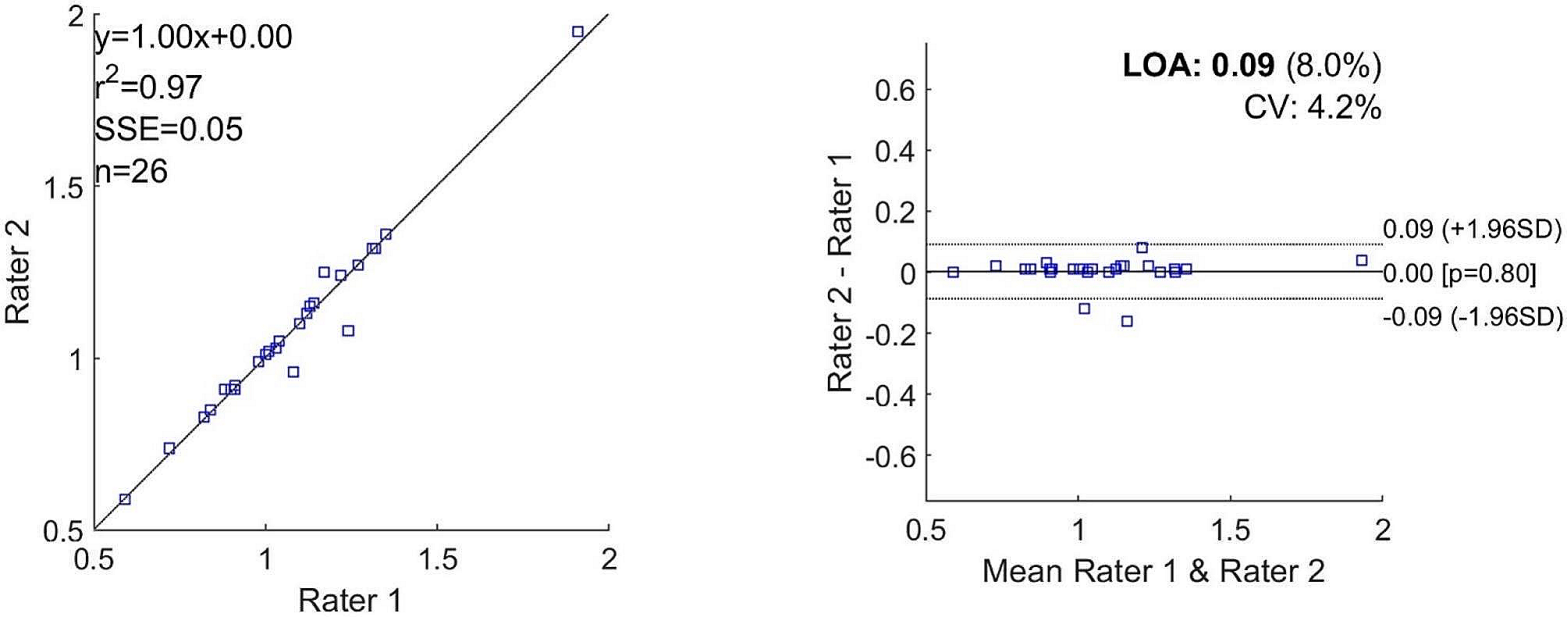
Bland-Altman plots and correlation plots for activity concentration at scan 1 Scan 1 activity concentration

**Fig. 3:**
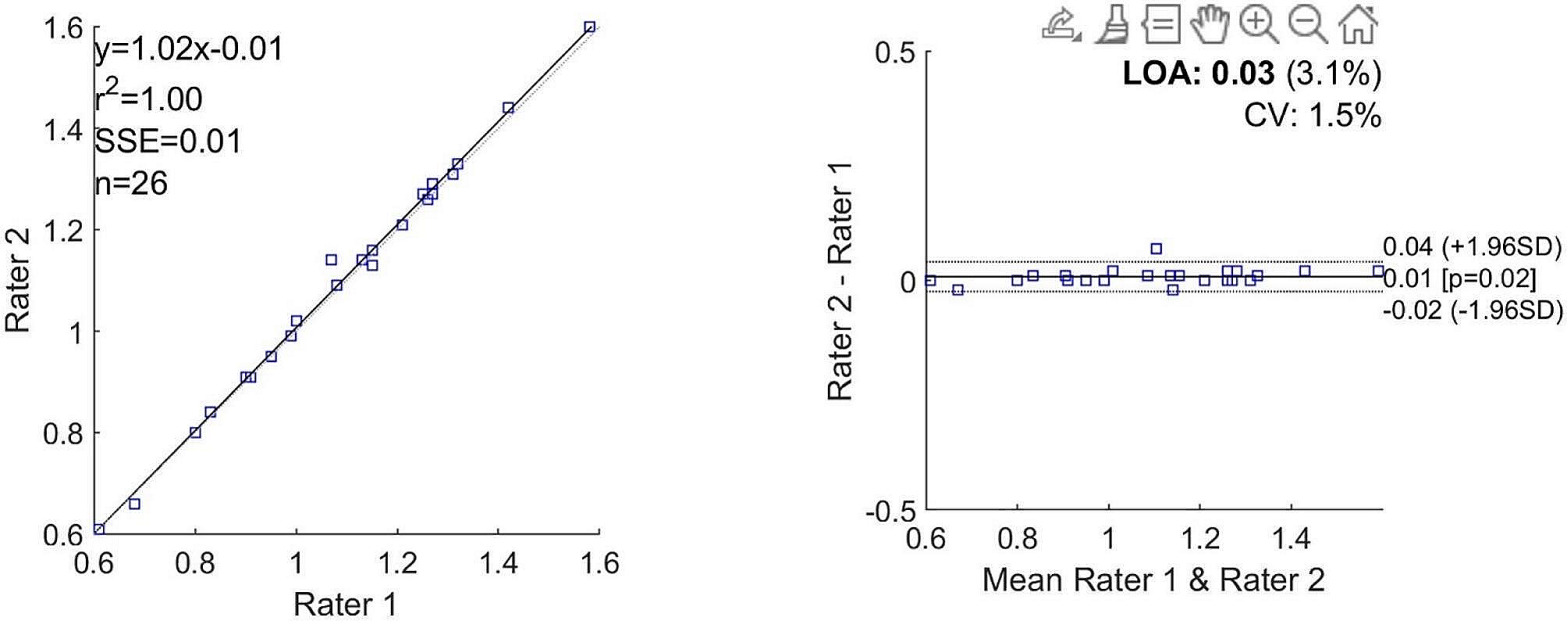
Bland-Altman plots and correlation plots for activity concentration at scan 2 Scan 2 activity concentration

Both raters calculated equal values for SUL (0.31 in the Liraglutide group and 0.26 in the placebo group) and for SUVbw (0.49 and 0.43, respectively in the Liraglutide and placebo groups). The values for TLG differed more as shown in Table 2. Bland-Altman analysis also showed good agreement between the two raters regarding activity concentration in the lungs (Figs. 2 and 3).

Inflammatory markers were measured in 30 completers (Liraglutide group, *n* = 17; placebo group, *n* = 13). Baseline concentrations of CRP and IL-6 were slightly elevated compared with normal ranges with median values of 3.69 and 4.37 mg/L for CRP and 5.05 and 4.38 pg/mL for IL-6 in the Liraglutide and placebo group, respectively. The normal ranges of inflammatory markers are given based on the laboratory’s normal ranges: CRP < 3 mg/L; MCP-1 = 72–295 pg/ml; IL-6 = 0.351–3.48 pg/ml (Table 4). Also MCP-1 levels were high in the normal range (72–295 pg/mL) with median values of 281 and 306 pg/mL in Liraglutide and placebo groups, respectively. We observed no between-group differences for median values of MCP-1, CRP, and IL-6 at baseline or after intervention at week 40 (Mann Whitney test). Further, we found no within-group changes in CRP, IL-6 and MCP-1 from baseline to week 40 in treatment or placebo groups (Table 4).

**Table 4:** Inflammation markers (median and 25–75 percentiles) at baseline and after 40 weeks in participants treated with Liraglutide or placebo

	Liraglutide (*n* = 17)			Placebo (*n* = 13)		
Variables	Baseline	40 weeks	*p*-value	Baseline	40 weeks	*p*-value
CRP (mg/L)	3.69 (2.83–5.24)	2.39 (1.69–4.47)	0.080	4.37 (2.42–6.91)	3.22 (1.79–8.30)	0.600
IL-6 (pg/mL)	5.05 (3.21–6.56)	4.03 (2.77–5.84)	0.278	4.38 (3.26–8.71)	4.81 (2.76–6.62)	0.347
MCP-1 (pg/mL)	281 (203–329)	283 (210–327)	0.586	306 (223–350)	274 (246–345)	0.507

CRP: C-reactive protein; IL-6: interleukin 6; MCP-1: monocyte chemoattractant protein-1

## Discussion

As part of a randomized clinical trial, 27 obese participants with COPD were scanned with FDG-PET/CT to quantify disease activity at baseline and after 40 weeks of treatment with Liraglutide 3.0 mg in terms of SUL, SUVbw, and TLG. We found no significant treatment effects for any of these parameters. As for plasma inflammation markers, we found no significant between-group effects and no changes from baseline to end of medication.

We compared our findings with uptake measures from three controls and two COPD patients with exacerbation. We found no difference in PET-based metabolic activity between project patients (Liraglutide and placebo) and controls. In patients with COPD exacerbation, we found higher values for tracer uptake resulting in higher values for SUL, SUVbw and TLG.

Bland-Altman plots showed that lung segmentation and the derived quantifications were reproducible.

Due to reported anti-inflammatory properties of GLP-1 RA, we expected the Liraglutide group to exhibit a reduction in systemic inflammatory markers. Similarly, we expected reduced PET-based metabolic activity in the lungs as a surrogate for inflammatory activity. Some previous studies on FDG-PET/CT found increased FDG lung uptake in COPD patients or current smokers compared to never-smokers as well as a correlation between FDG lung uptake and CRP. This inflammatory response in the airways with active neutrophils showed the potential of FDG as a surrogate marker of pulmonary inflammation [6]. Other studies found a correlation between metabolic activity in the intercostal accessory respiration muscles as a surrogate marker of COPD severity or increased FDG uptake in right ventricle indicating cor pulmonale secondary to pulmonary hypertension with increased severity of COPD [7].

However, we could not reproduce any of these findings in our study, for neither the inflammatory markers nor the PET-findings, and one explanation may be our study population; the inflammation markers were only slightly elevated or high in the normal range perhaps reflecting limited chronic disease activity. In addition, we measured inflammation markers in stable phases and not under exacerbations, where they are usually elevated. We cannot exclude statistical type II errors due to the relatively low number of study completers. Finally, differences in underlying methodology between the studies hamper direct comparison.

An indicator that the lack of positive findings in our primary study population may be due to overall low-level inflammation is the finding in the two patients with active COPD exacerbation, i.e. a tendency towards higher FDG-uptake in the lungs suggesting higher overall metabolism that may be due to generalized inflammation. However, motion artefacts may have influenced these results. Normal breathing motion causes motion blur artefacts in PET images. Ungated measurements may lead to falsely increased activity from liver activity measured as part of the lungs due to respiration motion. Our primary series were gated with limited impact from liver activity, but in the patients with COPD, the lack of gating may have influenced the overall lung activity. Respiration-gated PET approaches are employed to reduce the blurring effects in some clinical settings. In fact, others have found similarly equivocal results regarding whole lung quantification and proposed that the activity in whole lung may be too insensitive to detect lung inflammation at all [8]. To the best of our knowledge, there is no data on the reproducibility of respiration- gated segmentation in lung inflammation.

### Limitations

There might be different limitations in our study:

- The sample size is relatively small and furthermore the number of participants completing both scans were only 27 compared to the 40 patients included and randomized based on our power calculation.
- The study population may not have been be severely affected by COPD. We scanned the participants and measured circulating inflammation markers in stable phases of COPD, which might neglect a potential increase in inflammation in acute phases of the disease. A higher degree of disease burden in terms MRC dyspnea scale, eosinophils, number of exacerbations and the level of inflammatory markers under exacerbations could have affected the results positively, as indicated by the two COPD patients with exacerbation.
- The rationale for using respiration gating was an attempt to alleviate the potential effects of blurring from thoracic or abdominal movements from breathing which may cause a spillover of activity from the liver. The significance and impact of the methodology in this context remains unclear.
- The anti-inflammatory effect of Liraglutide also remains unconvincing in this setting and treatment with a more potent GLP-1 RA, eventually for a longer period, might have more anti-inflammatory effects.

## Conclusion

In contrast to other studies, we were not able to demonstrate differences in pulmonary inflammation using FDG-PET/CT in people with COPD before and after treatment with Liraglutide. With reference to the anti-inflammatory effects of Liraglutide and the promising role of FDG-PET/CT in the diagnosis of infectious and inflammatory diseases, we expected to find decreased uptake following treatment with Liraglutide. However, this was not the case. The inflammatory response may depend on the severity of COPD at the time of the scan (stable COPD versus exacerbation), and the patient population may simply have been in too stable stages. Based on our results, general application of FDG-PET/CT (with or without respiratory gating) in COPD cannot be recommended in relatively stable phases but whether FDG-PET-CT has a role in subset of COPD patients (e.g. more inflammatory active COPD or exacerbation) still needs further investigation.

## Data Availability

Datasets from the study are stored online in REDCap database. Data are available for the corresponding author and some of the other authors. Access to data is possible by contacting the authors. Some of the datasets, especially regarding FDG-PET/CT are also are also stored in AV Server regarding calculations about PET parameters. Data are also stored as written Case Report Forms (CRF) at the respective trial sites in locked rooms for 5 years. The CRF are checked and monitored by the Good Clinical Practice Unit at University of Southern Denmark.
